# Responsive Electrical Stimulation Suppresses Epileptic Seizures in Rats

**DOI:** 10.1371/journal.pone.0038141

**Published:** 2012-05-25

**Authors:** Lei Wang, Heng Guo, Xiao Yu, Shouyan Wang, Canhua Xu, Feng Fu, Xiaorong Jing, Hua Zhang, Xiuzhen Dong

**Affiliations:** 1 Faculty of Biomedical Engineering, the Fourth Military Medical University, Xi'an, China; 2 The 323rd Hospital of PLA, Xi'an, China; 3 Department of Neurosurgery, Tangdu Hospital, the Fourth Military Medical University, Xi'an, China; 4 Hearing and Balance Centre, Institute of Sound and Vibration Research, Southampton, United Kingdom; 5 Suzhou Institute of Biomedical Engineering and Technology, Chinese Academy of Sciences, Suzhou, China; Centre national de la recherche scientifique, France

## Abstract

**Background:**

A responsive electrical stimulation pattern based on our recently developed novel seizure prediction method was designed to suppress the penicillin-induced epileptic seizures.

**Methodology:**

Seizures were induced by Penicillin injection at rat cortex. A responsive electrical stimulation system was triggered prior to seizures predicted with phase synchronisation. Rats with induced seizures were stimulated by the electrical pulses at a responsive or 1 Hz periodic pattern of an open system. The effectiveness of stimulation on seizures suppression was assessed by measuring the average number and duration of seizures per hour.

**Results:**

The prediction algorithm reliably identified seizures in real time and triggered the responsive stimulation. This type of electrical stimulation dramatically suppressed seizure activity and the performance was better than the open stimulation system with fewer and shorter seizures.

**Conclusions:**

A responsive electrical stimulation system triggered by the phase synchronisation prediction is able to significantly suppress seizures.

**Significance:**

Responsive electrical stimulation could achieve superior treatment performance and reduce power consumption and side effects.

## Introduction

Treatment for epilepsy is still challenging in clinic. Pharmacologic treatments for epilepsy are safe but the effectiveness is not satisfactory [1]. Approximately one third of patients respond unfavorably to any antiepileptic medication or experience intolerable medication-related side effects [2]–[4]. For these intractable patients, surgical resection is an alternative treatment. However, the majority of patients with uncontrolled epilepsy will not have access to surgical therapy due to (1) high risk, i.e., a foci cannot be resected without damaging healthy tissue, resulting in permanent disability [5]–[6], (2) the inherently high technical complexity and cost, or (3) limited clinical and technical resources. Recently electrical brain stimulation has become available for movement disorders, pain and psychiatric diseases [7]. It has been used for epilepsy treatment as well although the performance is not as good as the treatment for movement disorders. Investigations in animals and humans have shown that electrical cortical stimulation can produce an inhibitory effect on seizures [8]–[13]. Brain stimulation has a much lower incidence of adverse cognitive, neurological, and systemic side-effects than that caused by anticonvulsant drugs [14].

Open-loop stimulation is delivered according to a predefined setting, independent of neurophysiological activity and/or brain activity. It usually is continuous stimulation at a given frequency. In order to improve the performance of brain stimulation, researchers have developed closed-loop or responsive stimulation according to varied principles. The closed-loop or responsive stimulation aims to suppress epileptic activity by delivering stimulation in response to the change of interictal neural activity [15]. The potential benefits of such stimulation include the ability to deliver therapy when epileptic activity occurs and the avoidance of side effects from anticonvulsants [16]. Implantable, local, closed-loop responsive neuro-stimulation systems represent a promising alternative treatment option in patients with well localised, focal, medically refractory epilepsy [17]. A responsive neurostimulation system (RNS) would limit the stimulus to the immediate preictal period, decreasing overall stimulus delivery over time and thus the likelihood of desensitisation and neuronal damage [18].

Gaito [19]–[20] has reported that low-frequency stimulation (1–3 Hz, LFS) results in strong and long lasting inhibition of epileptic activity induced by kindling. Since then, LFS for epilepsy suppression has been extensively studied both clinically and experimentally [21]–[22], but the effect of low-frequency responsive electric stimulation on epileptic focus or neural activity remains unclear. Moreover, most of closed-loop stimulation is based on the detection of seizure, which may delay the kick-in effect of stimulation. If the seizure is able to be predicted and the stimulation is delivered at the preictal period, such strategy should be able to further improve the efficacy of brain stimulation. This will also make it possible to record and stimulate at the same brain area.

Recently we developed one method to predict the occurrence of seizures, which is based on phase synchronisation in complex wavelet transform (PSW) [23]. The method achieved accuracy of 81.8%, i.e., correctly predicted 18 out of the 22 seizures in eight temporal epilepsy patients. The method was evaluated by the specificity (the ratio between the number of false predictions and the total observation time), seizure occurrence period (SOP, the period during which the seizure is to be expected) and seizure prediction horizon (SPH, the minimum window of time between the alarm raised by the prediction method and the beginning of SOP). These measures also confirm that the prediction based on phase of complex wavelet transform is a useful algorithm for predicting temporal lobe epilepsy on humans.

In this study, we aimed to develop a responsive cortical stimulation system according to the prediction of seizures based on phase synchronisation in rats. We tested the performance of the prediction algorithm on penicillin-induced epileptic seizures in rats. We evaluated the effects of low-frequency responsive electric stimulation on the epileptic seizures by the average number and duration of seizures per hour. The responsive electric stimulation was compared with the routine open loop stimulation and the non-stimulation group.

## Results

### Real time seizure prediction and EEG-guided stimulation

**Figure 1:**
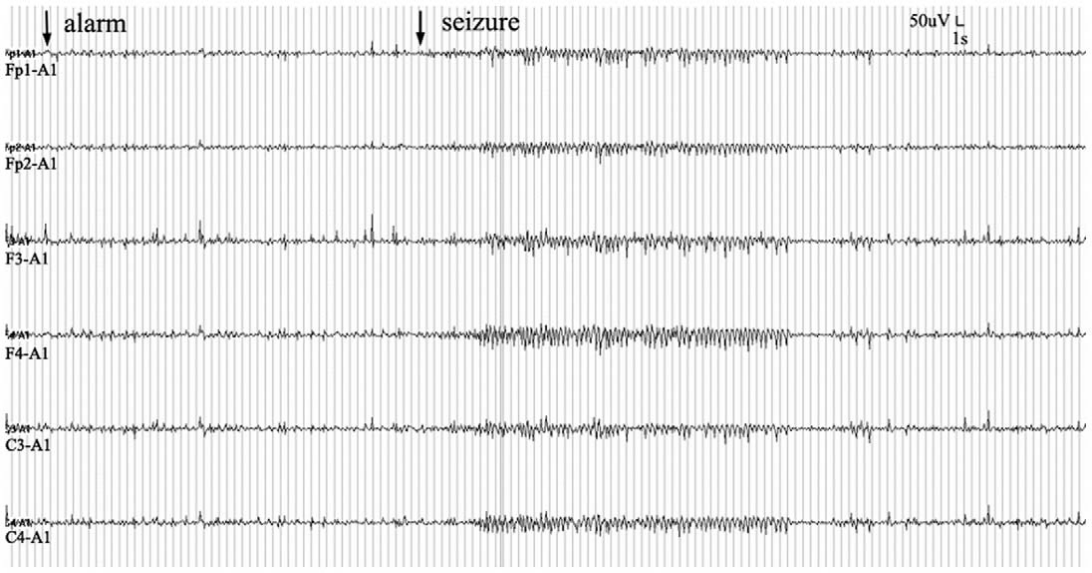
Simultaneously recorded EEG from Fp1, Fp2, F3, F4, C3 and C4 electrodes. Seizure was identified with phase synchronisation decreasing in two pairs of channels and marked by the second arrow. The phase synchronisation index raised an alarm about 49 seconds prior to the onset of the seizure.

Our EEG-guided responsive cortical stimulation system analyzed EEG in real-time, predicted seizures online, and triggered cortical stimulation in accordingly. The seizure monitoring was performed in non-stimulated and open-loop groups as well. A prediction alarming and seizure event is shown in Figure 1. The alarm occurred 49 s prior to the seizure. Effects of the prediction algorithm were evaluated in non-stimulation group. Overall sensitivity of PSW was 0.81 (198/244), false prediction rate was 0.29 per hour, and mean prediction time (time of true warning before a seizure) was 1.58±0.40 min. (Table 1).

**Figure 2:**
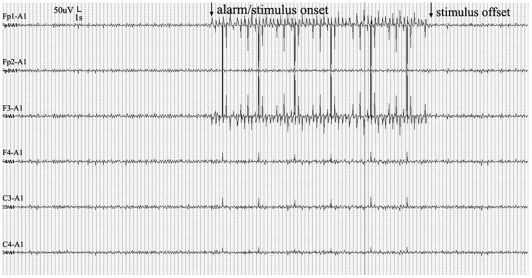
The responsive stimulation was triggered by reduced phase synchronisation. The cortical activity was continuously monitored.

Figure 2 illustrated a seizure prediction alarm in the responsive stimulation group (RSG) and activation of brain stimulation.

**Table 1:** Seizure prediction in non-stimulation group.

Animal	No. of Seizures	True predictions	False predictions	Sensitivity	Specificity (/hr)	Mean prediction time(min)
#1	48	41	3	0.85	0.43	2.16±0.61
#2	30	24	2	0.80	0.29	1.45±0.63
#3	31	23	1	0.74	0.14	1.26±0.50
#4	35	29	4	0.83	0.57	1.85±0.54
#5	37	29	1	0.78	0.14	1.03±0.34
#6	31	27	2	0.87	0.29	1.67±0.38
#7	32	25	1	0.78	0.14	1.36±0.40
Average	35	28	2	0.81	0.29	1.58±0.40

Sensitivity: the ratio between the correct predictions and the number of all registered seizures; Specificity: the ratio between the number of false predictions and the total observation time.

### Just-in-time seizure control

To quantify the effects of cortical stimulation on seizure suppression, the total number of seizures, the average duration of seizures and seizures per hour were quantified. The Statistical Program for the Social Sciences (SPSS12.0; IBM, USA) was used in statistical analysis. One-way ANOVA and post hoc Tukey's test were used for group comparisons. A p-value of less than 0.05 was considered statistically significant. A seizure was independently identified as continuous spike-wave discharge by experienced epileptologists.

**Figure 3:**
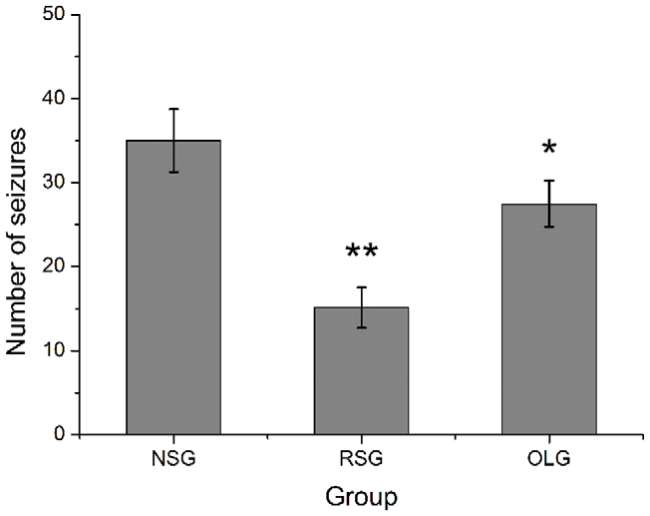
Effects of responsive and open-loop cortical stimulation on the occurrence of seizures. The number of seizures in 7 hours in RSG group was significantly lower than the NSG and OLG group. The responsive stimulation reduces seizures by about 56.6% and open loop stimulation reduces by about 21.3% compared with NSG group. Values are expressed as mean ± standard deviation (n = 7 in each group); **p<0.01 versus NSG; *P<0.05 versus NSG, one way ANOVA analysis; NSG: non-stimulation group; RSG: responsive stimulation group; OLG: open loop stimulation group.

There was significant difference between number of seizures of three groups (F = 11.21, P = 0.00, one-way ANOVA). Responsive cortical stimulation significantly decreased the number of penicillin-induced seizures. The average number of seizures in the RSG group was 15.14±6.39, significantly lower than the 27.43±7.3 observed in the open-loop stimulation group (OLG) (n = 7, post hoc Tukey's test, p = 0.02) and non-stimulation group (NSG) 34.86±6.31 (n = 7, post hoc Tukey's test, p = 0.00). Open-loop stimulation was also slightly effective, as the number of seizures was significantly lower than the NSG rats (n = 7, post hoc Tukey's test, p = 0.05) (Figure 3).

**Figure 4:**
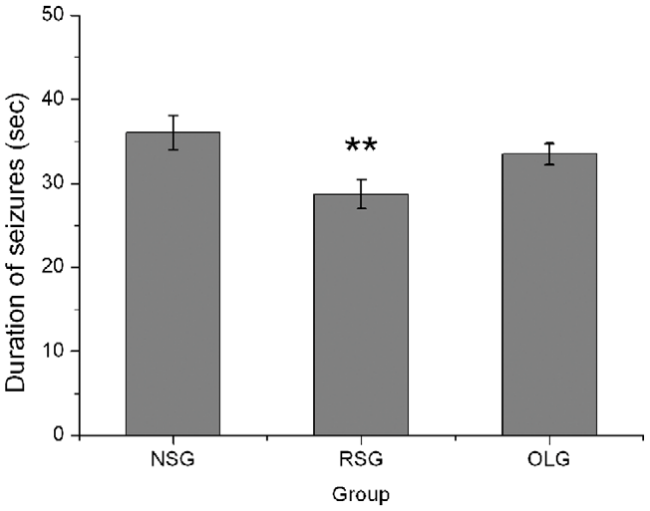
Effects of responsive and open-loop cortical stimulation on the average duration of seizures. The duration of seizures was significantly reduced in responsive stimulation group but not in open-loop stimulation group. Values are expressed as mean ±standard deviation (n = 7 in each group); **P<0.05 versus NSG group, one way ANOVA analysis. NSG: non-stimulation group; RSG: responsive stimulation group; OLG: open loop stimulation group.

The average duration of seizures of three groups were compared with one-way ANOVA, the difference between these three groups was significantly (F = 4.74, P = 0.02, one-way ANOVA). The average duration of seizures was also decreased significantly from 36.02±5.34 s in NSG group to 28.70±4.62 s in RSG group (n = 7, post hoc Tukey's test, P = 0.019). In contrast, seizure duration in NSG group did not differ significantly from OLG group (33.50±3.37 s) indicating that open-loop stimulation was ineffective in reducing seizure duration (n = 7, post hoc Tukey's test, p = 0.56) (Figure 4).

**Figure 5:**
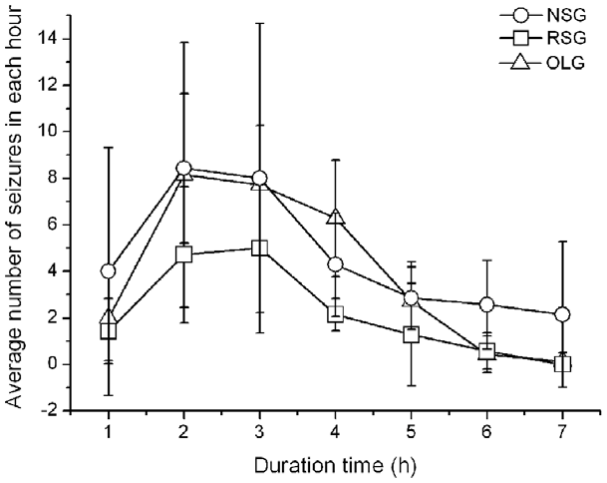
The occurrence of seizures after penicillin injection in three groups. The number of seizures in each hour was counted over seven hours after rats recovered. The occurrence of seizures changed over time. The seizures increased greatly after penicillin injection and reached maximum around the second and third hour and they decreased gradually thereafter. The number of seizures in RSG group are usually lower than the other two groups over seven hours and the peak value at the second or the third hour is only half of that in NSG or OLG group. NSG: non-stimulation group; RSG: responsive stimulation group; OLG: open loop stimulation group.

The average number of seizures per hour also indicated the effectiveness of responsive stimulation (Figure 5). Over time, the number of seizures per hour decreased in all three groups. The RSG group suffered seizures approximately half of that in NSG over whole period (Figure 5). We found a transient increase of seizure frequency in the first two hours after Penicillin injection.

## Discussion

Penicillin-induced focal epilepsy is a well-known model in experimental epilepsy [24]–[27]. Penicillin is a known gamma-aminobutyric acidA (GABA_A_) receptors antagonist, impairing the function of GABA-mediated inhibitory neurotransmission. Since the inhibition is impaired, recurrent excitatory postsynaptic potentials and intrinsic bursting of a subpopulation of pyramidal cells leads to an excessive cell firing in interconnected cortical neurons, and to a highly synchronized activity of the neuronal population [27]. In our study, there was no rat that died during the experiment. Generalized tonic-clonic seizures were observed within 20 to 30 min after penicillin injection, and epileptiform discharges was observed on the EEG during seizures.

In this study, a responsive electrical stimulation system with “just-in-time” automated seizure prediction was developed. This EEG-guided system integrated a phase synchronisation prediction method and electrical cortical stimulation techniques. It has reliably reduced the average number and duration of seizures. The performance of the prediction algorithm was evaluated on penicillin-induced seizures in non-stimulation control group. Sensitivity for prediction of seizures by the PSW algorithm was 81.1%, the average prediction time was 1.58±0.40 min. The average specificity of PSW for seizure prediction was 0.29 false warning per hour, or else about 7 false warnings per 24 hours. PSW studying phase synchronisation between different EEG channels could reflect any interactions between different regions of the brain and provide reasonable explanations with respect to electrophysiology. Otherwise, the wavelet transform possesses a few exceptional characteristics, making PSW suitable for predicting seizures. The performance of the responsive system was compared to that of an open-loop periodic stimulation control paradigm and a non-stimulation control group. Results showing responsive stimulation reduce seizure numbers 56.6% compared with NSG, and reduce seizure duration 20.3% compared with NSG. Responsive stimulation performs better than open-loop stimulation. The reason may be that closed-loop responsive stimulator would limit the stimulus to the immediate preictal period, decreasing overall stimulus delivery over time and thus the likelihood of desensitization and neuronal damage [18]. To date, the precise mechanism of action of closed-loop responsive electrical stimulation and how it suppresses seizures remain to be elucidated. One possibility is that the closed-loop control may be shunting energy at or near the focal area, thereby reducing excitability or action-potential amplitudes. Alternatively, the current injected could be altering the electrophysiologic dynamics of the neurons, thereby changing their firing patterns [1].

However, there are still some seizures remaining in the responsive stimulation group. Prediction algorithm and stimulation parameters are two key factors that affect the suppression effect of responsive stimulation. Unfortunately, at present, there is no one prediction algorithm that can predict correctly all preceding seizures. Furthermore, the optimal stimulation parameters are still unknown. Therefore, we need to improve prediction specificity and sensitivity, and determine the optimal stimulation parameters.

The ability of responsive stimulation to suppress seizures may depend on the stimulation parameters, including current intensity, stimulus duration, stimulus waveform, pulse frequency, and the timing and spatial location of the stimulation in relation to the spike discharge [28]. However, there are no general applicable optimal parameters to suppress seizures as different stimulation sites or different seizure types probably require different parameters. Even when the stimulation sites and seizure types are relatively constant, the results of therapeutic stimulation were still variable [11], [29]–[30].

In our study, we chose 1Hz square pulse electrical cortical stimulation with duration of 300 μs, and intensity of 0.1 mA to stimulate cortex. This low-frequency stimulation pattern suppressed seizures effectively, possibly because low-frequency electrical stimulation can polarize neuronal cells to modulate potassium spatial buffering [31] or yield a shift in somatic transmembrane potential and effectively suppress excitability [32]. Neocortical excitatory neurotransmission of synaptic can be short-term depressed by low-frequency electrical stimulation [33]. Furthermore, other nonneuronal cells that may be affected by electrical stimulation include endothelial cells that form the blood-brain barrier and tightly regulate the extracellular environment [34]. Finally, low-frequency stimulation could induce lasting changes in brain function [35] and foreshorten electrographic seizure duration in an acute seizure model [36].

Long-term electrical stimulation can induce neural injury. Animal studies suggested that the damage was correlated with charge density per phase and total charge per phase [37]. Charge density and charge per phase interact in a synergistic manner to determine the threshold of neural injury induced by electrical stimulation. The stimulus charge per phase is defined as the integral of the stimulus current over half (one phase) of one cycle of the stimulus. The usual units are millicoulombs or microcoulombs per phase (mC/ph or μC/ph). Charge density is defined as the integral of current density over either phase of the stimulus waveform. Its usual units are millicoulombs or microcoulombs per cm^2^ per phase (mC/cm^2^ per phase or μC/cm^2^ per phase) [38]. In our study, stimulus intensities, as expressed by stimulus charge per phase and charge density, were 0.03 μC/ph and 42.44 μC/cm^2^ per phase, a level that did not induce brain damage.

Thus, low-frequency stimulation could suppress seizures effectively while avoiding direct stimulus-induced damage to the neural tissue. Moreover, this model will help establish optimal parameters to analyze closed-loop responsive cortical electrical stimulation to suppress seizures. Thus, we will compare seizures suppression efficiency of responsive system using different stimulus parameters.

In contrast to treatment with antiepileptic drugs, the electrical stimulation on the brain can be directed preferentially targeted to one or several epileptic foci, to a specific pathway of seizure propagation, or to a particular structure that exerts more global modulatory effects, thus reducing adverse side effects. Responsive stimulation offers additional specificity and the treatment may be provided as needed, potentially reducing desensitization from periodic stimulation and the amount of antiepileptic drugs. In addition, a stimulus device has theoretical advantages over surgery because it is adaptable if seizures change and is reversible if functional disruption of the epileptogenic cortex causes adverse effects.

In this study, we have developed an effective responsive seizure control system which employed just in time (JIT) electrical stimulation. It can be more effective than an open-loop periodic stimulation system. This could become a highly effective and well-tolerated way of treating seizures, especially for patients with epilepsy of multifocal origin, or an origin that is difficult to locate. This study, as the first stage of work in progress, shows that it is feasible to close the loop between seizure prediction and brain stimulation for a better control of seizures. However, additional multi-institutional prospective clinical studies are required to evaluate the clinical efficacy of this novel treatment modality. Further technical improvements of this system along with the accumulation of clinical experience could lead to the development of an improved system that can predict seizures more accurately and abort them more efficiently.

## Materials and Methods

### Animals

All experimental procedures involving animals were conducted under a protocol reviewed and approved by the Ethics Committee of Tangdu Hospital, Fourth Military Medical University, Xi'an, China (approval ID: TDLL-2011071). Twenty-one adult male Sprague-Dawley rats weighing 280–320 g were used in this study. Rats were housed in an approved animal-care facility.

### Surgical procedures

**Figure 6:**
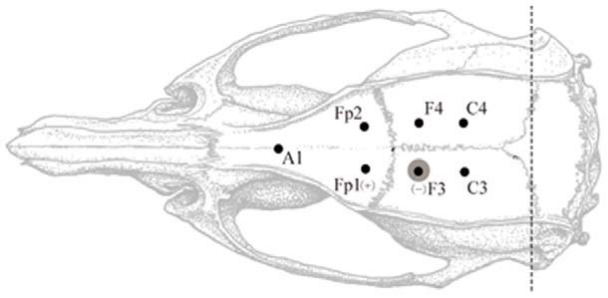
Diagram of placement of implanted electrodes. The penicillin is injected at area around F3 to induce epileptic seizures, which is marked by the grey area. The square wave electrical pulse are delivered between electrodes at Fp1(+) and F3(−). The EEG signals are simultaneously recorded from Fp1, Fp2, F3, F4, C3 and C4 electrodes against the reference electrode of A1.

Rats were anesthetized by 1% Pentobarbital Natricum (60 mg/kg) delivered intraperitoneally. After anesthesia was administrated, state of consciousness was regularly assessed by reaction to a toe pinch stimulus. Rats were attached to a stereotactic animal frame and a midline incision was made along the scalp to expose the skull. After holes were drilled into the skull, six stainless steel screw recording electrodes (diameter 0.3 mm) were placed epidurally 2 mm lateral to midline on both sides: two electrodes were placed over the frontal cortex, 2 mm anterior to bregma (Fp1 and Fp2); two electrodes were placed over the parietal cortex, 2 mm posterior to bregma (F3 and F4); and two electrodes were placed over the parietal cortex, 5 mm posterior to bregma (C3 and C4) (Figure 6). The reference electrode (A1) was placed epidurally 7 mm anterior to bregma at the midline. All electrodes except F3 were implanted and the connected wires were fixed respectively. Before the electrode F3 was implanted and connected with wire, penicillin (3 μl, 4I U/ml) was injected 2.3 mm below the skull at 1 μl/min through a micro syringe. The needle remained in place for 5 min after injection. Electrodes were fixed in place with dental resin. After the resin dried (several minutes), the scalp was sutured. Constant current stimulation was delivered by using the two electrodes Fp1 (anode) and F3 (cathode).

### Responsive cortical stimulation

#### Responsive cortical stimulation system

**Figure 7:**
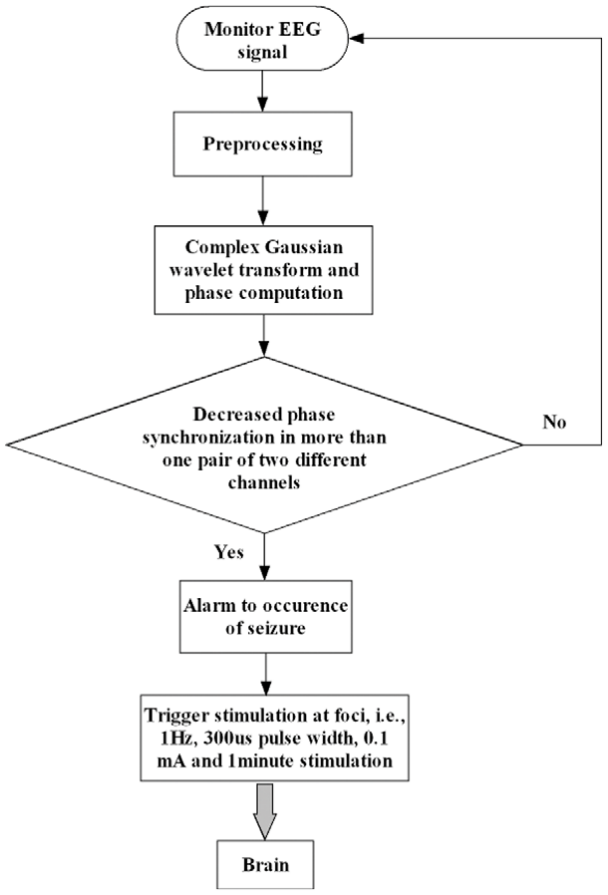
A Flow chart of the responsive stimulation system with automated seizure prediction and triggered stimulation.

The EEG-guided responsive cortical stimulation system consists of an electroencephalograms (EEGs) acquisition system, an automated seizure prediction and stimulation program, and a stimulator. The flow chart of the responsive stimulation system is depicted in Figure 7. EEGs were recorded with an electroencephalograph (NT9200-16V, SYMTOP INSTRUMNET Co., Ltd, China). The automated seizure prediction and stimulation program (ASPS) was an integration of prediction algorithm and acquisition software that predicted seizures in real time and triggered stimulator output if predicted alarm was raised. We introduced PSW as the prediction index. The necessary algorithm for seizure prediction and stimulation included the following methods: (1)The complex Gaussian wavelet transform was used to obtain the real part 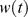 and imaginary part 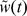 of EEG signals from every channel; (2)real part 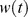 and imaginary part 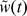 were used to determine the instantaneous phase of signals; (3)the phase difference 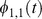 between two channels is then obtained; (4)phase synchronization index R was computed for every possible combination of different recording channels for the same consecutive window. (5) When more than seven continuous decreases in R in more than one pair of two different channels were detected, the prediction alarm is raised and the stimulator is triggered.

The ASPS program was developed on a visual studio 9.0 framework to operate in a Windows environment. The ASPS program triggered stimulator output when the ASPS detected a seizure occurring. Stimulation was given by a Nihon Kohden stimulator (Nihon Kohden, Japan) through a Nihon Kohden SS-202J constant-current stimulus isolation unit (Nihon Kohden, Japan).

#### Stimulation parameters

In this experiment, three groups of animals were used and they had the same placement of the epidural electrodes. They were randomly assigned to three groups, i.e., responsive stimulation group, open loop group and non-stimulation group, and each group had seven rats. The experiments were initiated only after the rats had completely recovered from anesthesia. EEG of all rats was then continuously monitored for 7 hours. In the RSG, animals were subjected to 1 min responsive cortical stimulation with 01 mA stimulus current, 300 μs pulse width, and 1 Hz stimulus frequency after an alarm. In the OLG, the stimulus with the same stimulation parameters as RSG, was given periodically at an interval (stimulus periodic interval, SPI) determined by RSG.
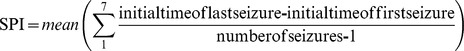
where, last seizure is the last one of seizures during recording time per rat in RSG and first seizure is the first one of seizures during recording time per rat in RSG. Number of seizures is all seizures observed during recording time in RSG.

Rats in the NSG were not given any stimulation.
