# Correction: Responsive Electrical Stimulation Suppresses Epileptic Seizures in Rats

**DOI:** 10.1371/annotation/1c4d82ab-6349-4ce8-b62b-43354df71c95

**Published:** 2012-08-20

**Authors:** Lei Wang, Heng Guo, Xiao Yu, Shouyan Wang, Canhua Xu, Feng Fu, Xiaorong Jing, Hua Zhang, Xiuzhen Dong

The first author, Lei Wang, should also be noted as contributing equally to this work. Authors Lei Wang, Heng Guo and Xiao Yu share joint first authorship.

